# Supplementary material for: The effect of librarian involvement on the quality of systematic reviews in dental medicine
Source: PLoS One. 2021 Sep 1;16(9):e0256833. doi: 10.1371/journal.pone.0256833 (PMC8409615; doi:10.1371/journal.pone.0256833)
Supplement: S1 Appendix — (DOCX) [file pone.0256833.s001.docx]

**S1 Appendix**

**Journals Included in Review and Number of Articles Examined**

Clinical Oral Implants Research – 140 results
Dental Materials – 45 results
European Journal of Oral Implantology – 40 results
International Endodontic Journal – 52 results
International Journal of Oral Science – 3 results
Journal of Clinical Periodontology – 190 results
Journal of Dental Research – 66 results
Journal of Dentistry – 141 results
Journal of Periodontology – 126 results
Monographs in oral science – 2 results
Oral Oncology – 101 results
Periodontology 2000 – 7 results
