# Supplementary material for: The effect of librarian involvement on the quality of systematic reviews in dental medicine
Source: PLoS One. 2021 Sep 1;16(9):e0256833. doi: 10.1371/journal.pone.0256833 (PMC8409615; doi:10.1371/journal.pone.0256833)
Supplement: S2 Appendix — (DOCX) [file pone.0256833.s002.docx]

**S2 Appendix**

**Search Strategy Used for This Review**

((systematic review [ti] OR meta-analysis [pt] OR meta-analysis [ti] OR systematic literature review [ti] OR this systematic review [tw] OR pooling project [tw] OR (systematic review [tiab] AND review [pt]) OR meta synthesis [ti] OR meta-analy*[ti] OR integrative review [tw] OR integrative research review [tw] OR rapid review [tw] OR umbrella review [tw] OR consensus development conference [pt] OR practice guideline [pt] OR drug class reviews [ti] OR cochrane database syst rev [ta] OR acp journal club [ta] OR health technol assess [ta] OR evid rep technol assess summ [ta] OR jbi database system rev implement rep [ta]) OR (clinical guideline [tw] AND management [tw]) OR ((evidence based[ti] OR evidence-based medicine [mh] OR best practice* [ti] OR evidence synthesis [tiab]) AND (review [pt] OR diseases category[mh] OR behavior and behavior mechanisms [mh] OR therapeutics [mh] OR evaluation studies[pt] OR validation studies[pt] OR guideline [pt] OR pmcbook)) OR ((systematic [tw] OR systematically [tw] OR critical [tiab] OR (study selection [tw]) OR (predetermined [tw] OR inclusion [tw] AND criteri* [tw]) OR exclusion criteri* [tw] OR main outcome measures [tw] OR standard of care [tw] OR standards of care [tw]) AND (survey [tiab] OR surveys [tiab] OR overview* [tw] OR review [tiab] OR reviews [tiab] OR search* [tw] OR handsearch [tw] OR analysis [ti] OR critique [tiab] OR appraisal [tw] OR (reduction [tw]AND (risk [mh] OR risk [tw]) AND (death OR recurrence))) AND (literature [tiab] OR articles [tiab] OR publications [tiab] OR publication [tiab] OR bibliography [tiab] OR bibliographies [tiab] OR published [tiab] OR pooled data [tw] OR unpublished [tw] OR citation [tw] OR citations [tw] OR database [tiab] OR internet [tiab] OR textbooks [tiab] OR references [tw] OR scales [tw] OR papers [tw] OR datasets [tw] OR trials [tiab] OR meta-analy* [tw] OR (clinical [tiab] AND studies [tiab]) OR treatment outcome [mh] OR treatment outcome [tw] OR pmcbook)) NOT (letter [pt] OR newspaper article [pt]))

AND

("Clin Oral Implants Res"[Journal] OR "Dent Mater"[Journal] OR "Eur J Oral Implantol"[Journal] OR "Int Endod J"[Journal] OR "Int J Oral Sci"[Journal] OR "J Clin Periodontol"[Journal] OR "J Dent"[Journal] OR "J Dent Res"[Journal] OR "J Periodontol"[Journal] OR "Monogr Oral Sci"[Journal] OR "Oral Oncol"[Journal] OR "Periodontol 2000"[Journal])
