# Supplementary material for: The effect of librarian involvement on the quality of systematic reviews in dental medicine
Source: PLoS One. 2021 Sep 1;16(9):e0256833. doi: 10.1371/journal.pone.0256833 (PMC8409615; doi:10.1371/journal.pone.0256833)
Supplement: S3 Appendix — (DOCX) [file pone.0256833.s003.docx]

**S3 Appendix**

**Questions Included in First Round of Analysis (Questionnaire 1)**

1. Who is the reviewer?
2. Unique ID of the Article
3. Is there librarian participation? *Also consider information specialist, informationist, etc. as librarian.
4. Where is search information located?
5. Is the search reproducible?
6. Did the authors list their inclusion criteria?
7. Did the authors list their exclusion criteria?
8. Blinding?
9. Do the authors report the number of people who reviewed the full text of the initially included articles?
10. How many people reviewed the full text of initially included articles?
11. Do the authors report the number of people who reviewed the titles/abstracts in the review?
12. How many people reviewed the titles/abstracts in the review?
13. Did the authors provide the number of studies found?
14. Did the authors provide the number of duplicates removed?
15. Did the authors provide the number of titles/abstracts screened?
16. Did the authors provide the number of full text articles reviewed?
17. Did the authors provide the number of articles fully included?
18. Was a risk of bias assessment performed for the articles that passed initial full text screening?
