# Supplementary material for: The effect of librarian involvement on the quality of systematic reviews in dental medicine
Source: PLoS One. 2021 Sep 1;16(9):e0256833. doi: 10.1371/journal.pone.0256833 (PMC8409615; doi:10.1371/journal.pone.0256833)
Supplement: S4 Appendix — (DOCX) [file pone.0256833.s004.docx]

**S4 Appendix**

**Questions Included in Second Round of Analysis (Questionnaire 2)**

1. Who is the reviewer?
2. Unique ID of the Article
3. Is the search strategy reproducible
4. How many databases were searched?
5. Which databases were searched?
6. Did the authors conduct a grey lit search?
7. Which database is the primary reported search strategy mapped for?
8. Is the search strategy reproducible exactly as written? (tags/filters included in strategy so that all that is needed is copying/pasting)
9. Is the search strategy reproducible with minor manipulation?
10. The search strategy is translated well into concepts.
11. Are there any mistakes in the use of parentheses or brackets?
12. Are there any mistakes in the use of Boolean operators?
13. Are there any mistakes in the use of proximity operators?
14. Are important subject headings missing? (Please note: check the date on MeSH entries against the date of the review before selecting 'yes')
15. Have irrelevant subject headings been included?
16. Are any natural language terms missing?
17. Are any natural language spelling variants missing?
18. Have any irrelevant natural language terms been included?
19. Is truncation used optimally?
20. Does the search strategy have any spelling mistakes?
21. Does the search strategy have system syntax errors?
22. Does the search strategy have wrong line numbers?
23. Do any of the limits used seem unwarranted?
