# Supplementary material for: The effect of librarian involvement on the quality of systematic reviews in dental medicine
Source: PLoS One. 2021 Sep 1;16(9):e0256833. doi: 10.1371/journal.pone.0256833 (PMC8409615; doi:10.1371/journal.pone.0256833)
Supplement: Attachment — Submitted filename: Response to Reviewers 2.docx [file pone.0256833.s006.docx]

Plos ONE Rebuttal Document – Version 2

We again appreciate the thoughtful comments made by Reviewer 2. We appreciate the time and effort taken to help us improve this paper for publication. We believe these adjustments have significantly improved the paper. Thank you.

1. The authors do not discuss the role of librarian authorship in the introduction specifically, yet it is one of the primary research questions. Perhaps it would be helpful to add a brief sentence describing why this would be important in the intro?
The authors have added additional content to the introduction, but I am not seeing any addition of information specifically about librarian co-authorship. I still think it would be helpful to mention something specifically about authorship prior to the research questions. The same is true with grey literature, which is a research question, but is not specifically addressed in the intro.

Added wording about Gray Literature to lines 76-77 and wording about co-authors to lines 82-87 (including a reference [29] to the ICMJE recommendations for authorship).

2. Line 97: The authors clearly conducted their search prior to the new revision of the PubMed systematic reviews subset search. It might be helpful to note that this was the active query at the date of the search; it is completely different today.
This item was unfortunately written poorly by me. I meant that the systematic review subset search has changed, not PubMed (though that is also true). If the authors compare the current PubMed systematic reviews search with the old one, they will see it has significantly changed. However, with the date included, it will help readers understand that this was the subset search at the time of that search. Unfortunately, NLM doesn’t seem to track versions of this search anywhere that I’ve seen.

Added wording to lines 113-114 about the use of the Clinical Queries search string prior to the PubMed update. Also, complete search is included in Appendix A, with the Clinical Queries search contained in the full search.

3. Lines 146-48: How did the authors conduct their analysis? What statistics were planned to be used? What were the planned analyses (i.e., multivariate regression? Linear regression?)? I see one location (line 173) where the authors mention one statistical analysis used, but this is the only place. For example, see the primary comparative articles that are similar topics for their analytical methods: Koffel et al, Meert et al, Rethlefsen et al.
I appreciate the additional detail provided by the authors about their analyses. I think there is still more work to be done here. “Mostly” should be avoided. What variables were tested using chi squares, e.g.

Added wording to Data Analysis section (lines 193-204) and Table 1

4. It would be helpful to have other information about the data set in the results; information about years included, number of reviews per journal title, etc, could potentially help readers get a better grasp on what is actually being studied. AUTHOR RESPONSE: Unfortunately, we do not have data about number of reviews per journal. Added language to data analysis section for more clarity.
I think the authors do have this information, or I would hope so? Do you not have the citations for the included studies?

As all studies were identified with PMID numbers, this was not a data point we originally ran. Re-ran the journal title search compared to all included PMID numbers in response to this comment. Added number of examined articles from each journal to Appendix A.

5. Discussion section: There is no comparison with other research anywhere in the Discussion section, which makes it hard to understand the context of these findings. Do they differ from other disciplines? Do they match other (non-librarians’) studies done in dental medicine? I would really like to see an expanded discussion section here, as I think this is an important study that would benefit from contextualization.
A few new comments:
6. The authors very nicely lay out new research questions. The associated detail with those questions, however, should be in the methods section. Lines 93-6; 99-101

Moved associated detail to the methods section.
7. PRISMA 2009, PRISMA 2020, and PRISMA-S are mentioned in several places in the text, but the latter two are not cited. It would be appropriate to add citations. (Lines 397; 432-3)

Added references for PRISMA 2020 and PRISMA-S
8. I could be missing something, but it looks to me like the information in lines 272-5 is redundant with the information in the paragraph above—with one major caveat. The caveat is that, in line 270, I think 71% is supposed to be 74%.

Adjusted language for clarity. Now lines 273-284
9. Lines 307-310: The authors may want to consider moving this up to be their first data point in the results, as the analyses do all seem to depend upon it.

We have arranged the paper with two main Results sections: Reporting in All Reviews; Analysis of Reproducible Searches. We have arranged the data point as the first in the section labeled: Analysis of Reproducible Searches.
